# Supplementary material for: Species pool and soil properties in mangrove habitats influence the species-immigration process of diazotrophic communities across southern China
Source: mSystems. 2024 Jul 9;9(8):e00307-24. doi: 10.1128/msystems.00307-24 (PMC11334429; doi:10.1128/msystems.00307-24)
Supplement: Supplemental Material — s and methods, figures, and tables. [file msystems.00307-24-s0001.docx]

**Supplementary** **Materials for**

**Species pool and soil properties in mangrove habitats influence the species-immigration process of diazotrophic communities across southern China**

Nengjian Liao *et al*.

*Corresponding author. Email: nli0417@163.com

**This PDF file includes:**

1. Materials and methods
2. Figs. S1 to S12
3. Table S1 to S2

**Materials and methods**

**Study area and field sampling**

The sampling site was located in coastal mangrove wetlands along a latitudinal gradient transect, spanning a wide geographic range (18.44°–24.39°N and 108.24°–117.92°E) (Fig. 1a). In July 2019, we collected 600 individual samples of *Kandelia obovata* from six accessible natural mangrove areas in southern China (two sites in Hainan (LD and DZ), two sites in Guangxi (ZZ and SK), one site in Guangdong (HD), and one site in Fujian (FG). Five plots (5 × 5 m^2^) were established for sampling in each region. The samples were fractionated into four components, with 150 samples per type (Fig. 1B): nonrhizosphere soil (NS), rhizosphere soil (RS), root endosphere (RE), and plant leaves (LE), which were selected as models to elucidate the underlying immigration mechanisms of the diazotrophic communities from the soil to plant leaves. The nonrhizosphere soil was shaken off the roots, whereas the rhizosphere soil (~ 1 mm thickness around the root) was washed off with sterile water. The clean roots were subsequently washed three times to remove the remaining soil and placed in 1 × TE buffer supplemented with 0.1% Triton X-100 in a 50 mL Falcon tube. The plant leaf litter of *K. obovata* was immediately washed with sterile water, freeze-dried, weighed, and ground. All samples were stored at −80 °C until DNA was extracted.

**Environmental properties measurements**

For soils in the nonrhizosphere, soil properties, including oxidation–reduction potential (ORP), salinity, temperature, and pH, were measured using a pH and ORP meter (HI98121; Hanna, Italy). Soil texture (sand, silt, and clay) was determined using a Malvern Mastersizer 2000 (Malvern, United Kingdom) (Christensen and Olesen, 1998). Nutrients, including total carbon (TC), total organic carbon (TOC), total nitrogen (TN), and total sulfur (TS), were determined using an elemental analyzer (Vario Macro Cube, Germany). The total inorganic carbon (TIC) was defined as TC minus TOC. Total phosphorus (TP) was measured as previously described by Wang et al. (1). Soil water extracts were used to measure SO_4_^2–^ and PO_4_^3–^ via ion chromatography (ICS-2100; Dionex, USA). Inorganic nitrogen (NO_2_^–^-N, NO_3_^–^-N, and NH_4_^+^-N) was extracted using 2 M KCl and determined using a continuous-flow analyzer (SAN++; Skalar, Breda, the Netherlands). Biotic factors (total plant coverage, *K. obovata* number, *K. obovata* average height, *K. obovata* basal diameter, *K. obovata* canopy area, *K. obovata* coverage) were estimated in 5 × 5 m plots during the field survey. In addition, *K. obovata* aboveground biomass, *K. obovata* belowground biomass, and *K. obovata* total biomass were calculated using allometric equations by Rahman et al. (2). Data on climatic factors, including the mean annual temperature, mean annual precipitation, mean annual evaporation, mean annual relative humidity, and mean annual sunshine duration, were collected from the China Meteorological Data Sharing Service System (<http://data.cma.cn/>) (Table S1).

**DNA extraction and PCR amplification**

Approximately 0.25 g of frozen soil samples were used for DNA extraction using a Power Soil DNA Kit (MoBio, Carlsbad, CA, USA). Approximately 0.05 g of root endosphere samples were extracted using a Power Plant Pro DNA Isolation Kit (Mo Bio Laboratories, Inc., Carlsbad, CA, USA) after thorough grinding in liquid nitrogen. Plant leaf samples were washed to remove epiphytic diazotrophs. Plant leaf DNA was extracted using a GeneJET Genomic DNA Purification Kit (Thermo Scientific, Waltham, USA) (3). The DNA concentration was measured using a NanoDrop 1000 spectrophotometer (Thermo Scientific, Wilmington, MA, USA). The functional gene nifH was amplified using nifH-F (5ʹ-AAAGGYGGWATCGGYAARTCCACCAC-3ʹ) and nifH-R (5ʹ-TTGTTSGCSGCRTACATSGCCATCAT-3ʹ) primers to investigate the diazotrophic community (4). Each 20 μL reaction mixture comprised 0.25 μL (5 units) of EX Taq (TaKaRa, Dalian, China), 0.2 μL of each primer (20 μM), 1 μL template DNA (20–30 ng), and 18.35 μL of sterile distilled water. The PCR amplification conditions were as follows: initial denaturation of 95°C for 3 min, followed by 30 cycles of 94°C for 1 min, 55°C for 1 min, elongation at 72°C for 60 s, and a final extension step of 72°C for 10 min. Finally, the PCR product was recovered using an EZNA Gel Extraction Kit (Omega Bio-tek, Norcross, GA, USA). The DNA concentration was measured using a NanoDrop 1000 spectrophotometer (Thermo Scientific, Wilmington, MA, USA). High-throughput sequencing of the *nifH* gene was conducted on an Illumina MiSeq platform using a 2 × 250 bp Reagent Kit v2.

**Bioinformatics analysis**

Raw sequences were analyzed to remove sequences with primer mismatches or lengths of < 275 bp, low-quality reads (quality scores < 30), and barcode sequences. Chimera filtering and trimmomatic quality control were performed using the DADA2 denoising method in the QIIME2 (5). High-quality sequences were combined and grouped into amplicon sequence variants (ASVs) without imposing an arbitrary dissimilarity threshold (6). Taxonomic assignment was based on both the nucleotide and translated amino acid sequences (6). The original sequence data obtained from our research have been submitted to the National Center for Biotechnology Information (NCBI) Sequence Read Archive (SRA) database under BioProject Accession PRJNA770021.

**Data analysis**

The relative abundance-weighted community degree (RACD) was calculated to examine the variations of diazotrophic community associations by different components, as follows: ∑N×RA, where N is the number of ASVs, and RA is the relative abundance of the ASVs(7). RACD values represent the degree of close association among ASVs in different components of the diazotrophic community. To compare the immigration processes of diazotrophic communities across the different components, we divided the sample into four groups: NS, RS, RE, and LE. The immigration process of NS→RS was defined as the diazotrophs from NS to RS, while the immigration process of RS→RE was defined as the diazotrophs from RS to RE, and the immigration process of RE→LE was defined as the diazotrophs from RE to LE. In this study, Simm is the number of immigrating ASVs (e.g., in the immigration process from NS to RS, Simm indicates ASVs detected only in NS), Sext is the number of extinct ASVs (ASVs lost from the previous sample), and Stot is the total number of ASVs from both samples involved in the immigration process. The overall change in the species composition was measured using the richness-based species exchange ratio (SERr), which was quantified as follows: SERr = (Simm + Sext)/Stot (8). We also partitioned SERr into the species extinction ratio (SExR) and species immigration ratio (SImR), defined as Simm/Stot and Sext/Stot, respectively (7). Before calculation, all samples were rarefied to the same sequence for downstream analysis.

The alpha diversity of the diazotrophic communities in different components was determined by calculating the Shannon index. Beta-diversity was indicated using nonmetric multidimensional scaling (NMDS) based on the Bray–Curtis dissimilarity, and analysis of similarity (ANOSIM) between diazotrophic communities in different components was performed by the “vegan” package (9). The species pool of each sample was estimated using the “specpool” function in the “vegan” package (10). Nonparametric testing (KwWlx) and Wilcoxon tests were performed to reveal the differences using the “EasyStat” and “stats” packages, respectively. The UpSet plot was used to visualize using online software ([https://www.cloudtutu.com](https://www.cloudtutu.com/%23/index)) to represent the number of common and unique ASVs in the different components (NS, RS, RE, and LE). Mantel test analysis was used to further identify the driving factors explaining variations and assembly mechanism in the diazotrophic community using the “vegan” package. We calculated the β-nearest taxon index (βNTI) to further infer the assembly processes of diazotrophic communities (11). βNTI > 2 indicates heterogeneous selection of a deterministic process, βNTI < −2 indicates homogeneous selection of the deterministic process, whereas βNTI values between −2 and 2 indicates that the community assembly was dominated by stochastic processes. Then, community assembly was further distinguished using the modified Raup-Crick index (RC_Bray_) within a certain group and chemical property ranges with 999 randomizations, which is able to indicate dispersal limitation and homogeneous dispersal of the stochastic processes with RC_Bray_ > 0.95 and |βNTI| < 2, RC_Bray_ < −0.95 and |βNTI| < 2, respectively. The fraction of pairwise comparisons with |βNTI| < 2 and |RC_Bray_| < 0.95 was used to estimate the influence of the “non-dominant” fraction (i.e., ecological drift) (11). Variation partitioning analysis (VPA) was conducted to examine the contribution of biotic and abiotic factors to the community assembly according to the RDA (12). Linear regression analysis was used to examine the potential relationships between geographical distance and various parameters, including diversity measures (alpha diversity and beta diversity), species pool, RACD, SERr, SExR, and SImR. Additionally, the model analysis explored the effects of diversity (alpha diversity and beta diversity) and species pool on the immigration process (SERr, SExR, and SImR). The shared ASVs among diazotrophic communities from the four distinct components were defined as Groups I–IV (Group I: ASVs shared between NS and RS; Group II: ASVs shared between NS, RS, and RE; Group III: ASVs shared between NS, RS, RE, and LE; Group IV: ASVs shared between NS and LE), as previously described by Zhang et al. (13). We classified the environmental properties into four groups: soil properties, nutrients, climate factors, and biotic factors to evaluate their influence on the SERr, SExR and SImR using Pearson’s correlation test in the “vegan” and “dplyr” packages. Partial least squares path modelling (PLS-PM) was conducted to determine the direct and indirect contributions of soil properties, nutrients, climate factors, and biotic factors, alpha diversity, beta diversity, species pool on the SImR using the “plspm” package.

**Supplementary Figures.**

**
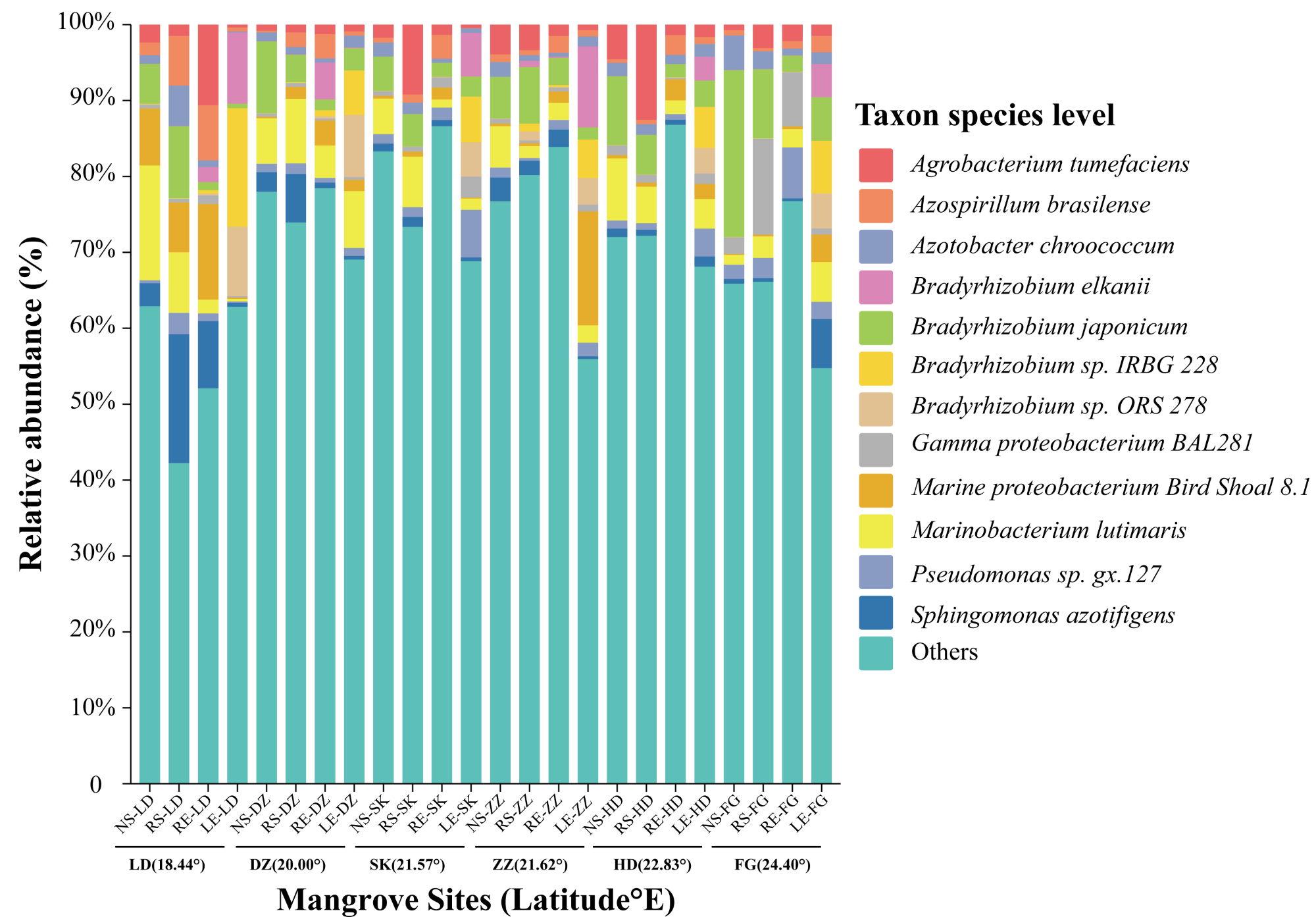
**

**Fig. S1 Relative abundance analysis of community composition of the top 12 species.** LD: Ledong mangrove site; DZ: Dongzhaigang mangrove site; SK: Shankou mangrove site; ZZ: Zhenzhuwan mangrove site; HD: Huidong mangrove site; FG: Fugong mangrove site. NS, nonrhizosphere soil; RS, rhizosphere soil; RE, root endosphere; LE, leaves.

**
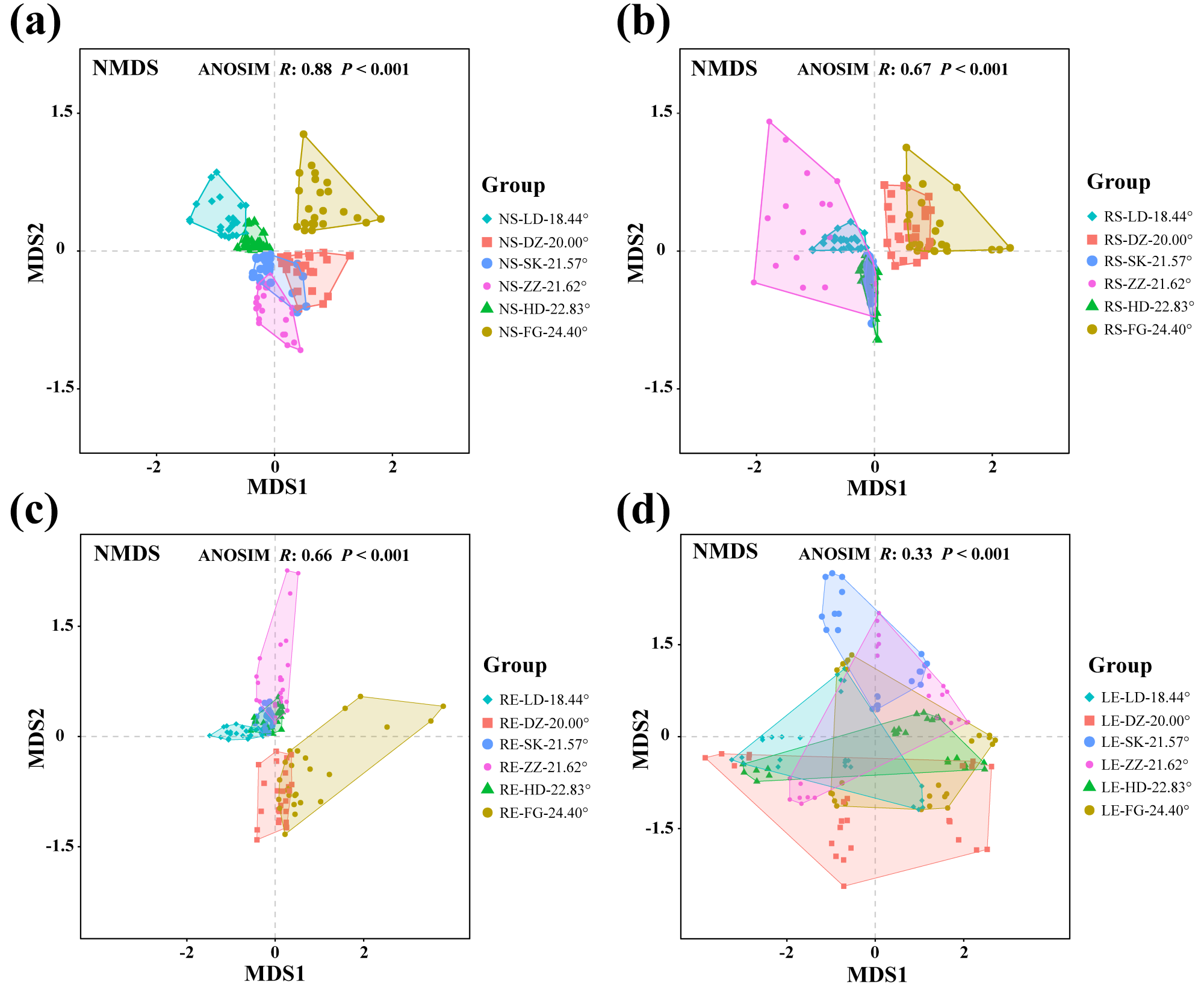
**

**Fig. S2 The beta diversity was calculated based on Bray–Curtis dissimilarity and visualized using nonmetric multidimensional scaling (NMDS) of different sites and components. (a)**: NS group in six mangrove sites; **(b)**: RS group in six mangrove sites; **(c)**: RE group in six mangrove sites; **(d)**: LE group in six mangrove sites. LD: Ledong mangrove site; DZ: Dongzhaigang mangrove site; SK: Shankou mangrove site; ZZ: Zhenzhuwan mangrove site; HD: Huidong mangrove site; FG: Fugong mangrove site. NS, nonrhizosphere soil; RS, rhizosphere soil; RE, root endosphere; LE, leaves.


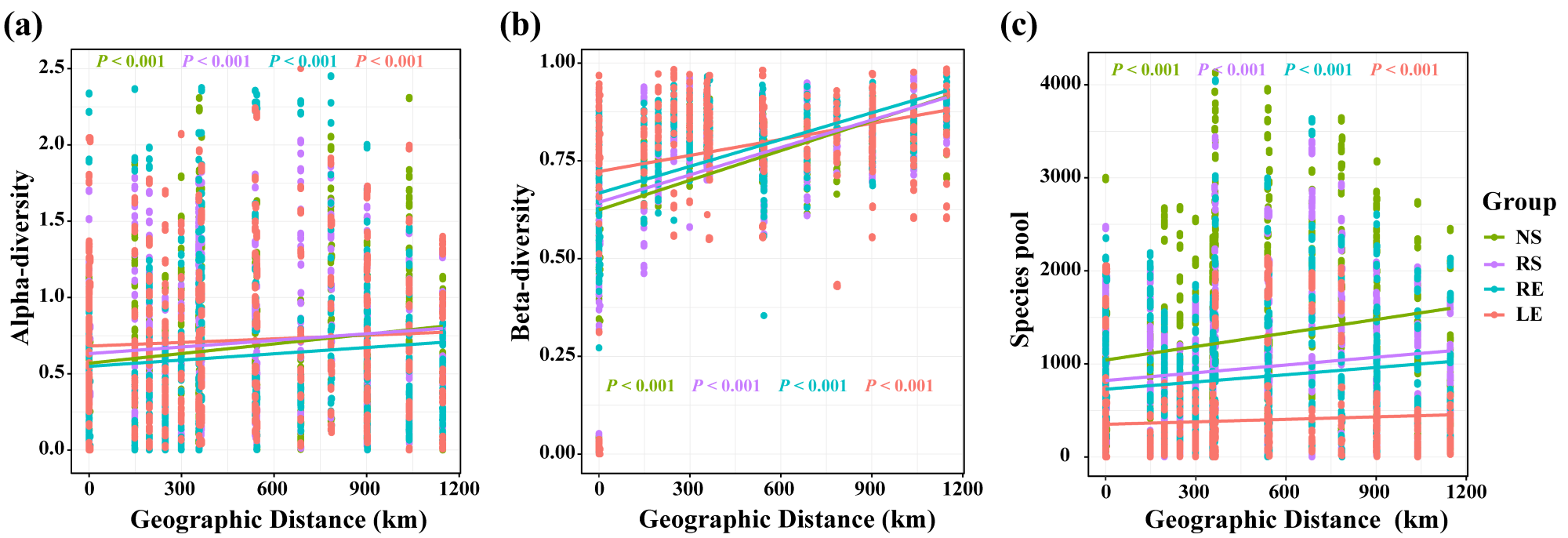


**Fig. S3 Linear regressions for geographic distance associated with alpha diversity (a), beta diversity (b), and species pool (c) of the diazotrophic communities.** NS: nonrhizosphere sample; RS: rhizosphere sample; RE: root endosphere sample; LE: leaves sample.


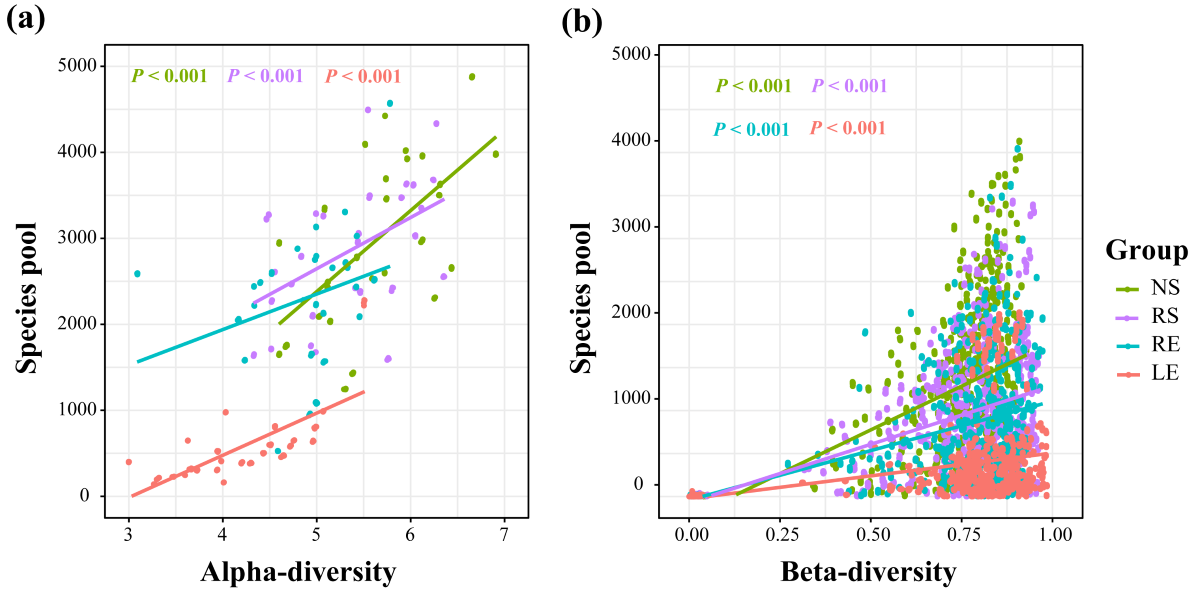


**Fig. S4 Linear regressions for alpha- (a) and beta-diversity (b) associated with species pool of the diazotrophic communities.** NS: nonrhizosphere sample; RS: rhizosphere sample; RE: root endosphere sample; LE: leaves sample.


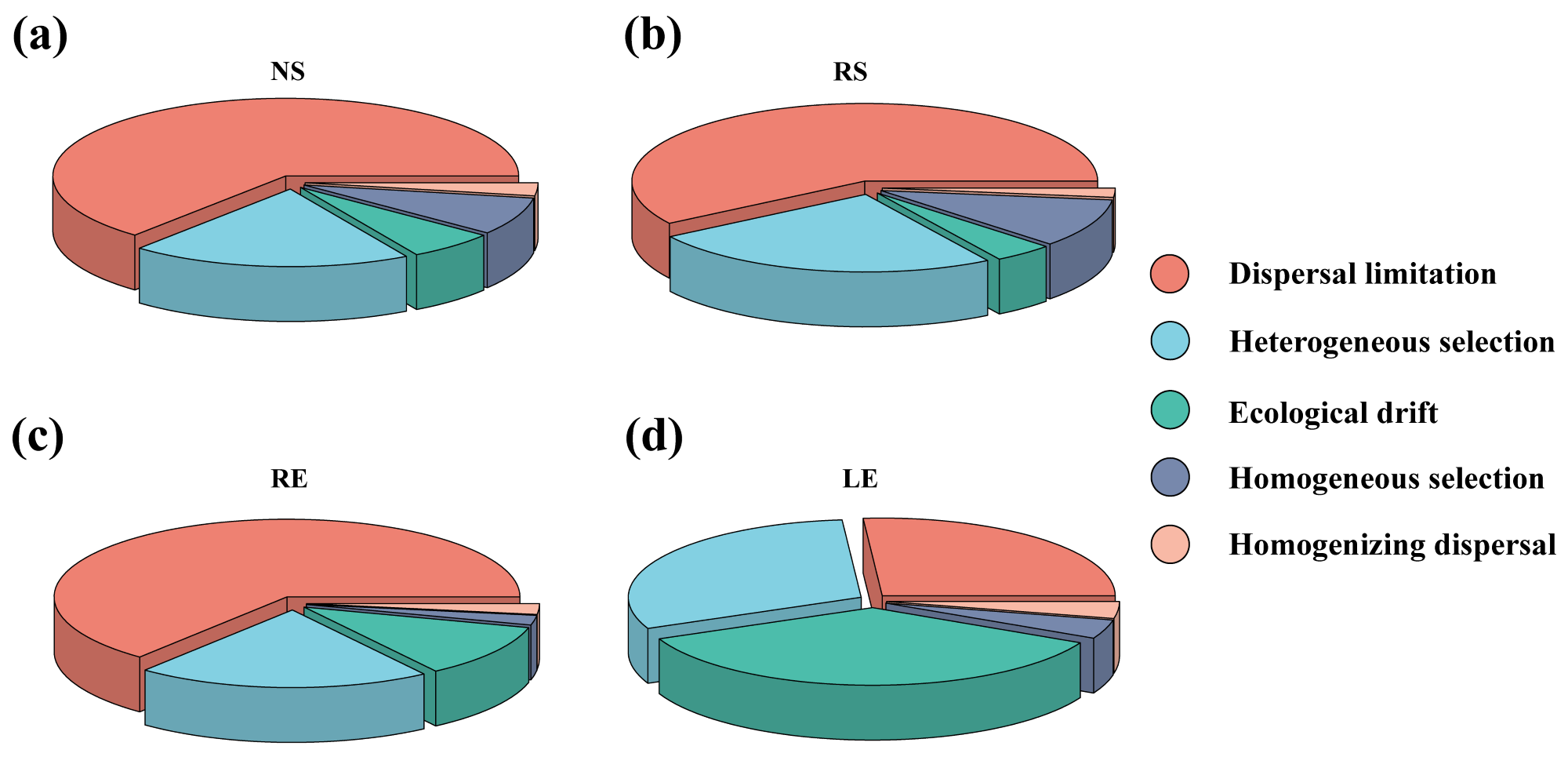


**Fig. S5 The pie plots display the diazotrophic community assembly with NS (a), RS (b), RE (c), and LE (d) in different components at large-scale mangrove sites.** NS: nonrhizosphere sample; RS: rhizosphere sample; RE: root endosphere sample; LE: leaves sample.


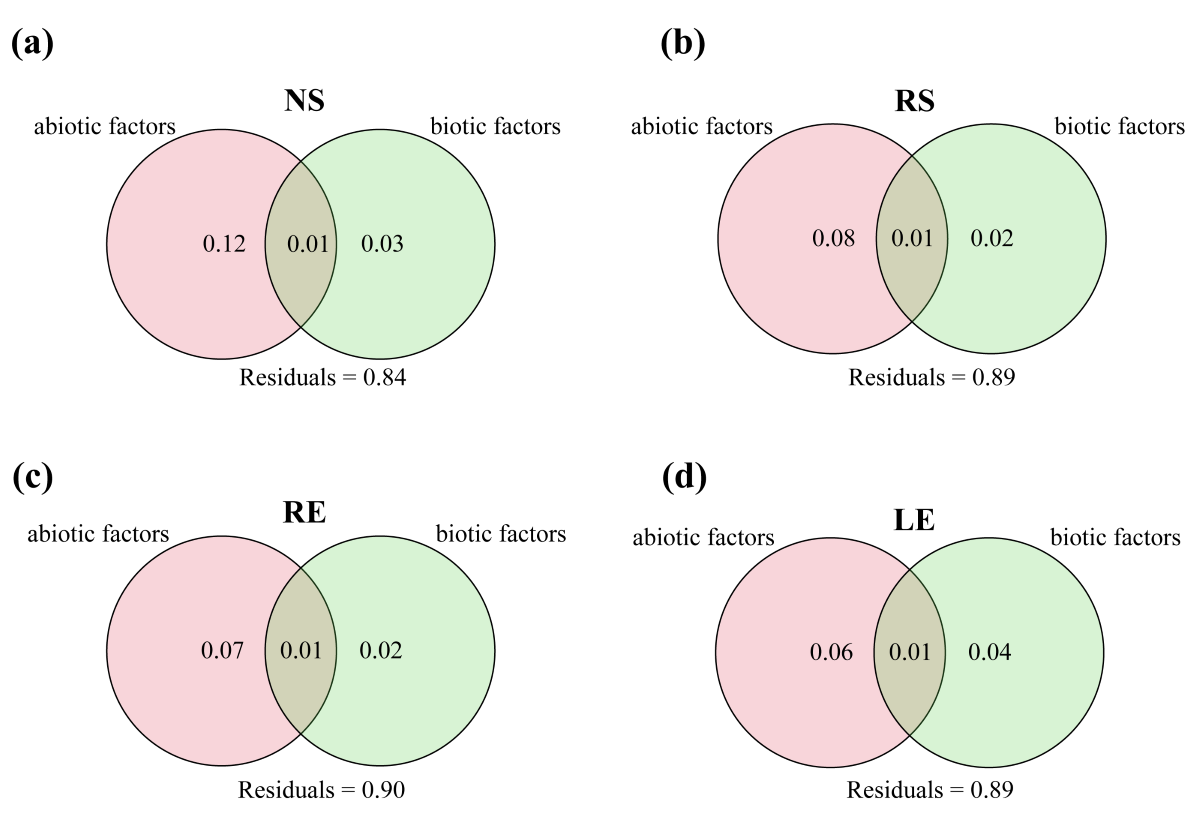


**Fig. S6** Variation partitioning analysis shows the effects of abiotic factors (ORP, pH, temperature, salinity, clay, silt, sand, TS, SO_4_^2-^, TP, PO_4_^3-^, TN, TC, TOC, TIC, NO_2_^-^-N, NO_3_^-^-N, NH_4_^+^-N, MAT, MAP, MAE, MAR, and MAS) and biotic factors (PTC, KN, KAH, KBD, KCA, KC, KAB, KBB, and KTB) on the βNTI of diazotrophic community. NS: nonrhizosphere sample; RS: rhizosphere sample; RE: root endosphere sample; LE: leaves sample. ORP: Oxidation reduction potential; TS: Total sulphur; TP: Total phosphorus; TN: Total nitrogen; TC: Total carbon; TOC: Total organic carbon; TIC: Total inorganic carbon; MAT: Mean annual temperature; MAP: Mean annual precipitation; MAE: Mean annual evaporation; MAR: Mean annual relative humidity; MAS: Mean annual sunshine; PTC: Plant total coverage; KN: *Kandelia obovata* numbers; KAH: *Kandelia obovata* average height; KBD: *Kandelia obovata* basal diameter; KCA: *Kandelia obovata* canopy area; KC: *Kandelia obovata* coverage; KAB: *Kandelia obovata* aboveground biomass; KBB: *Kandelia obovata* belowground biomass; KTB: *Kandelia obovata* total biomass.


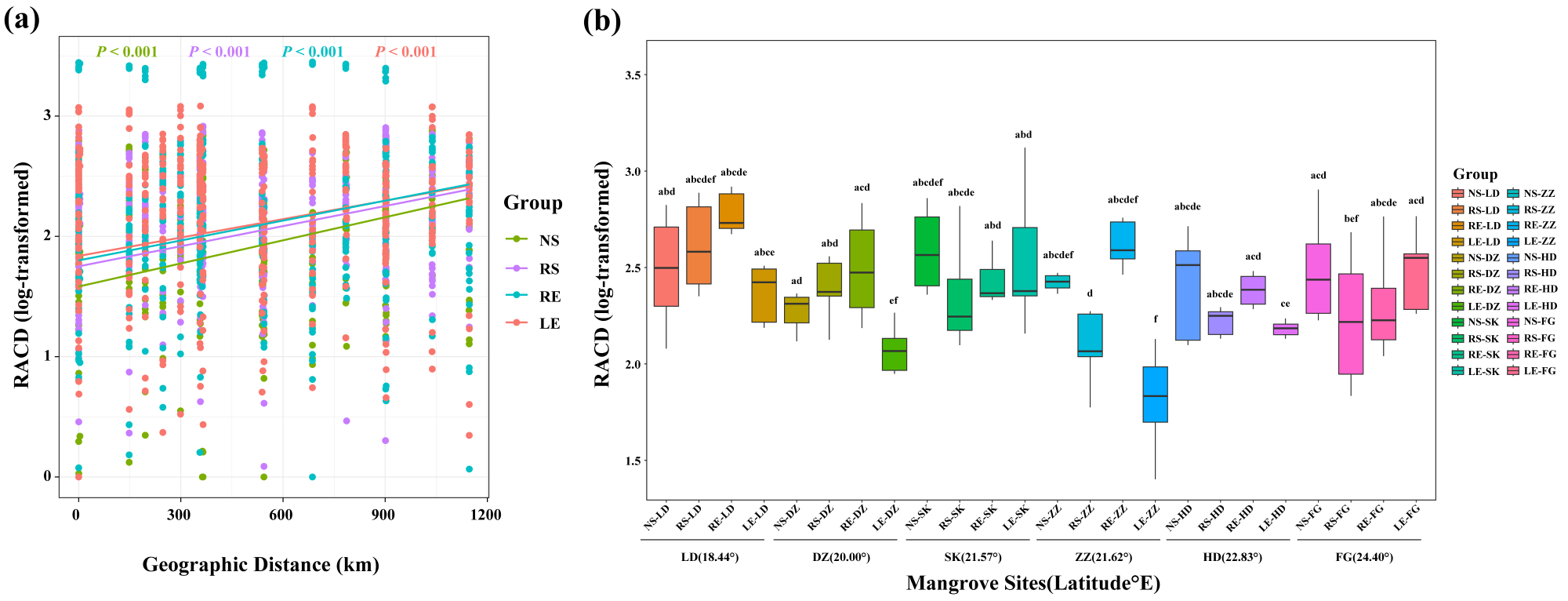


**Fig. S7 Linear regression for geographic distance associated with log-transformed RACD (a) of the diazotrophic communities in different components. The log-transformed RACD** **(b) in different groups along latitude gradient were presented by boxplot.** The different letters above the box plot indicate a signiﬁcant difference at *P* < 0.05. NS: nonrhizosphere sample; RS: rhizosphere sample; RE: root endosphere sample; LE: leaves sample.


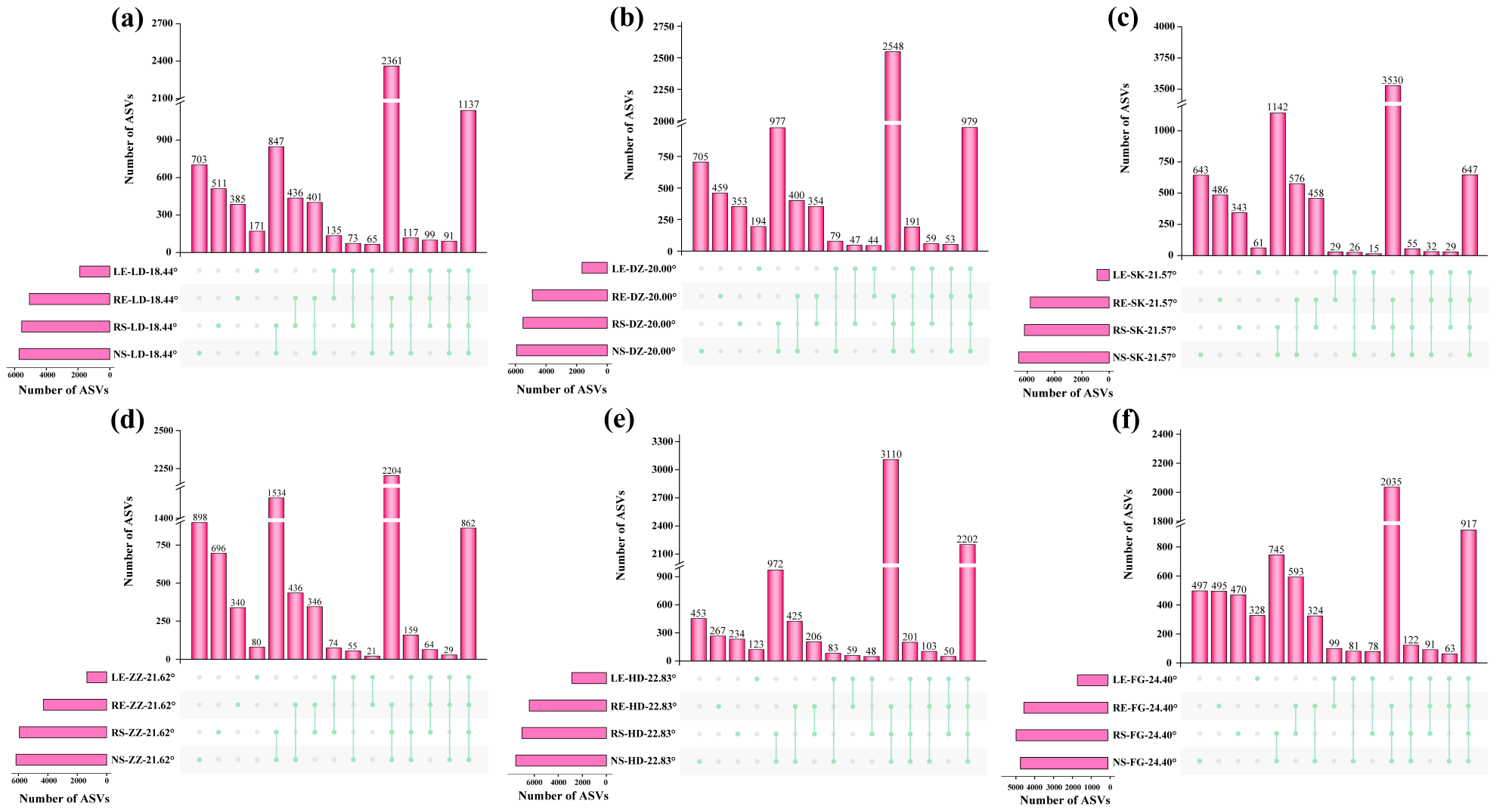


**Fig. S8 The UpSet plot visualizes the common and unique ASVs in different sites and components.** **(a)**: Ledong (LD) mangrove site; **(b)**: Dongzhai (DZ) mangrove site; **(c)**: Shankou (SK) mangrove site; **(d)**: Zhenzhuwan (ZZ) mangrove site; **(e)**: Huidong (HD) mangrove site; **(f)**: Fugong (FG) mangrove site. NS: nonrhizosphere sample; RS: rhizosphere sample; RE: root endosphere sample; LE: leaves sample.


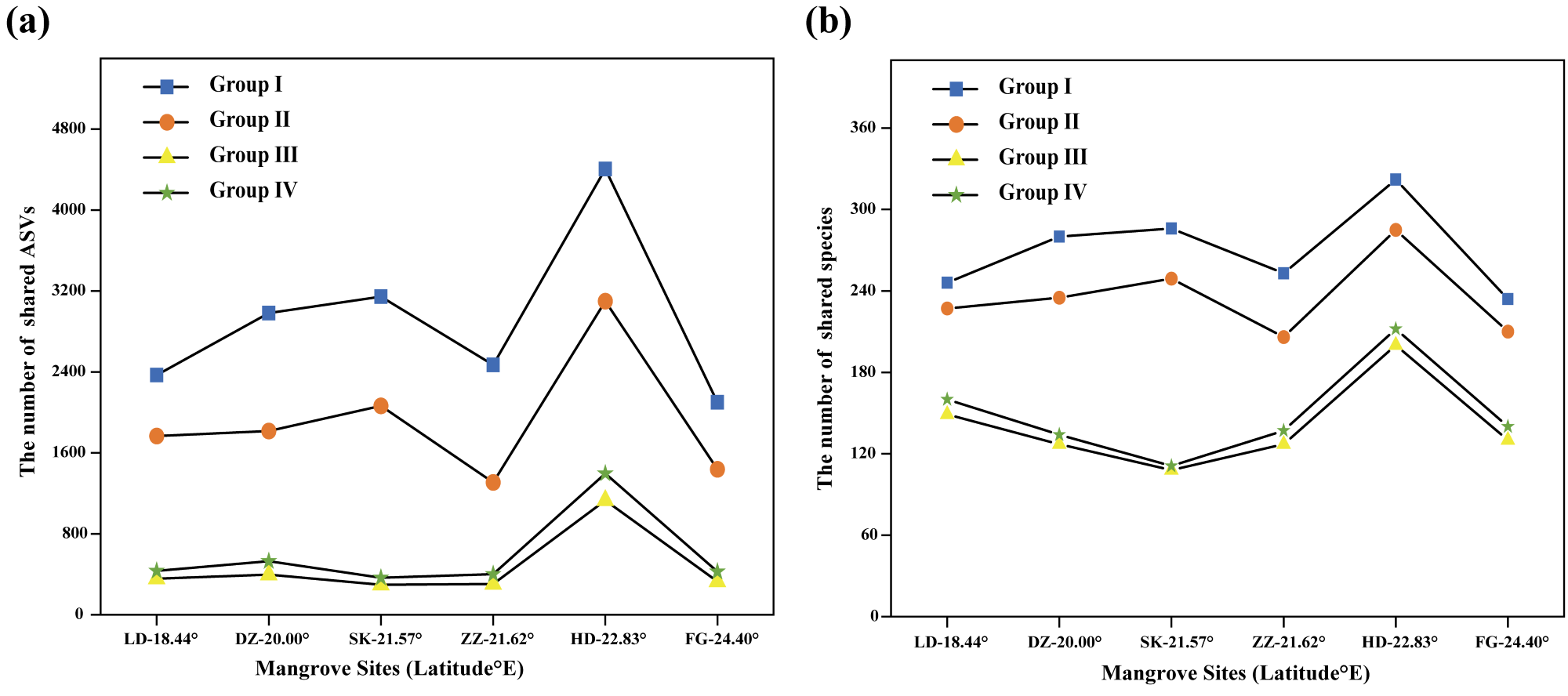


**Fig. S9 The variation in the number of shared ASVs (a) and species (b) along latitude gradients in different mangrove sites.** Group Ⅰ indicated the ASVs shared between NS and RS; Group Ⅱ indicated the ASVs shared between NS, RS, and RE; Group Ⅲ indicated the ASVs shared between NS, RS, RE, and LE; Group Ⅳ indicated the ASVs shared between NS and LE. LD: Ledong mangrove site; DZ: Dongzhaigang mangrove site; SK: Shankou mangrove site; ZZ: Zhenzhuwan mangrove site; HD: Huidong mangrove site; FG: Fugong mangrove site. NS: nonrhizosphere sample; RS: rhizosphere sample; RE: root endosphere sample; LE: leaves sample.


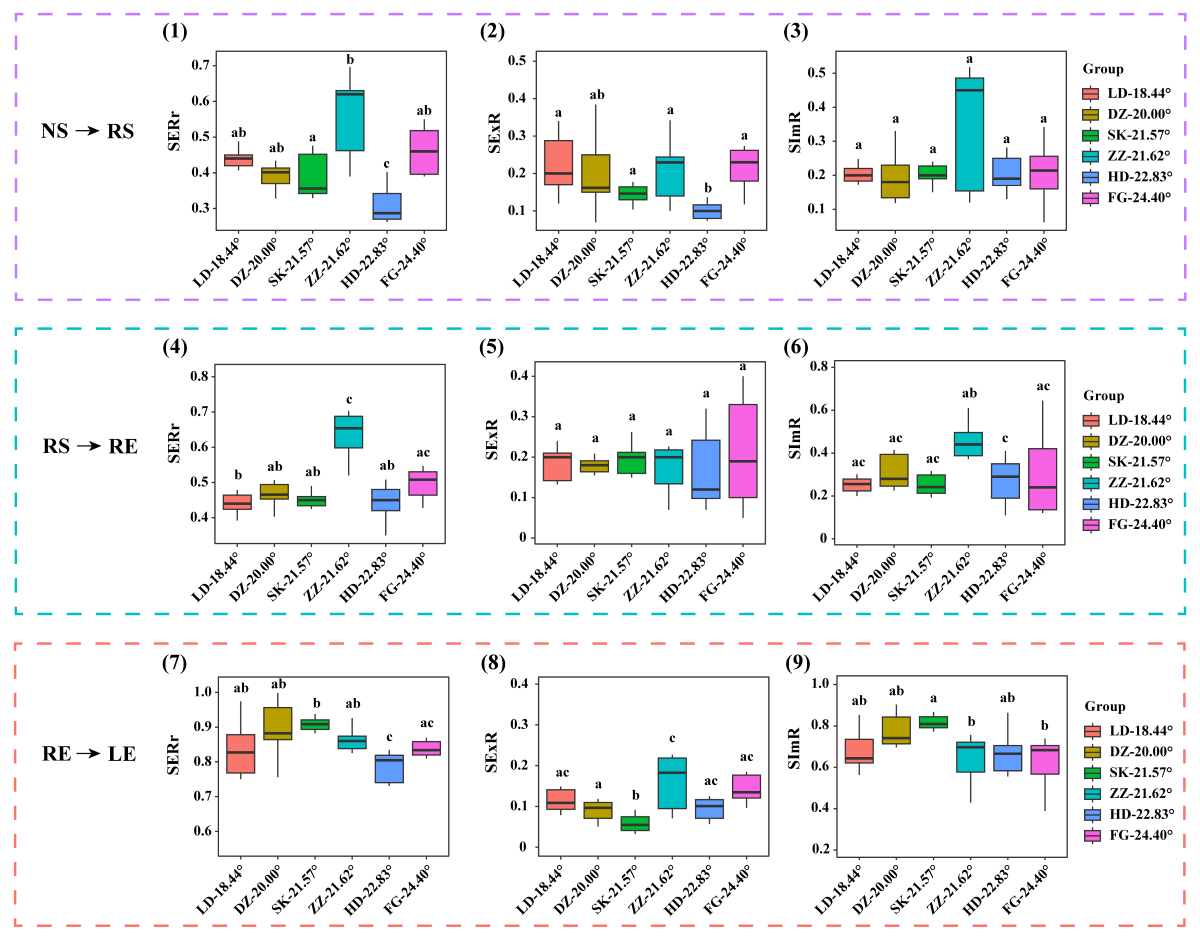


**Fig. S10 The** **variation in immigration mechanisms of diazotrophic communities by boxplot.** The values of species-exchange ratio (SERr) **(1)**, **(4)**, **(7)**, species-extinction ratio (SExR) **(2)**, **(5)**, **(8)**, and species-immigration ratio (SImR) **(3)**, **(6)**, **(9)** in different mangrove sites along latitude gradient. Different letters above the box plot on the indicated a significant difference (*P* < 0.05) using the Wilcoxon test. NS→RS, the transmission pathway from nonrhizosphere soil to rhizosphere soil; RS→RE, the transmission pathway from rhizosphere soil to root endosphere; RE→LE, the transmission pathway from root endosphere to leaves. LD: Ledong mangrove site; DZ: Dongzhaigang mangrove site; SK: Shankou mangrove site; ZZ: Zhenzhuwan mangrove site; HD: Huidong mangrove site; FG: Fugong mangrove site. NS: nonrhizosphere sample; RS: rhizosphere sample; RE: root endosphere sample; LE: leaves sample.


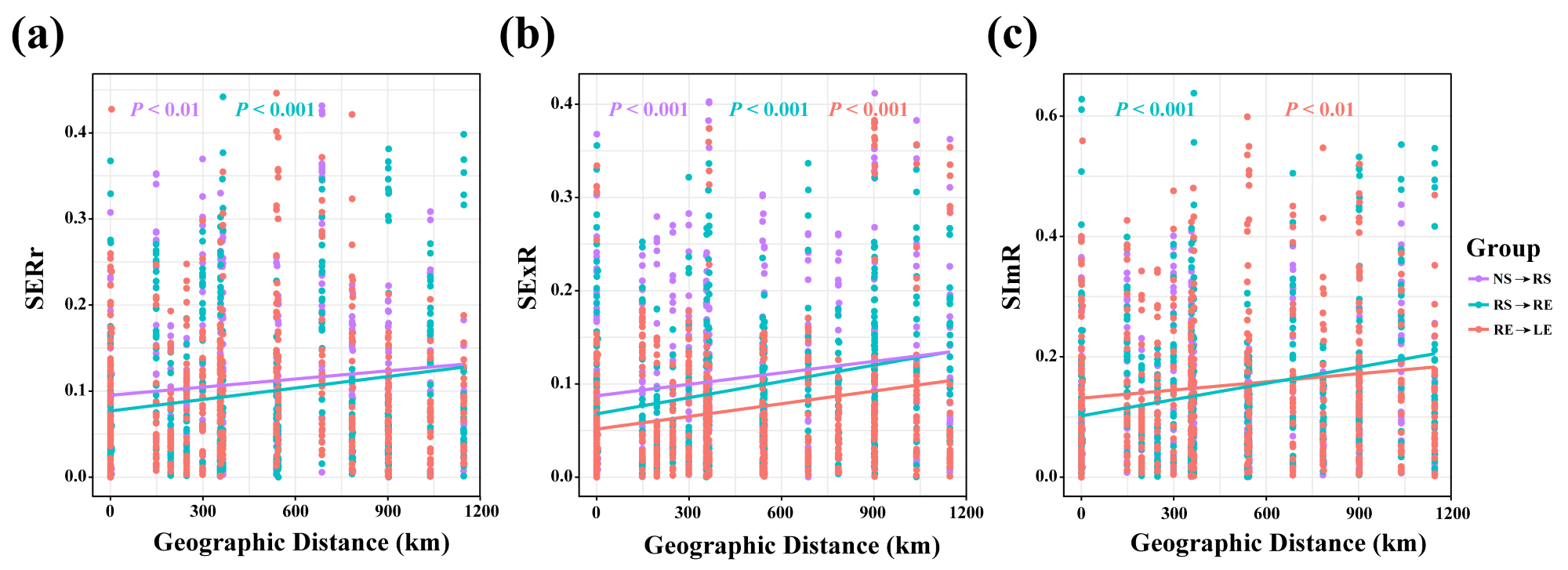


**Fig. S11 Linear regression for geographic distance associated with SERr (a), SExR (b), and SImR (c) in different components of the diazotrophic communities.** NS→RS, the transmission pathway from nonrhizosphere soil to rhizosphere soil; RS→RE, the transmission pathway from rhizosphere soil to root endosphere; RE→LE, the transmission pathway from root endosphere to leaves. NS: nonrhizosphere sample; RS: rhizosphere sample; RE: root endosphere sample; LE: leaves sample.


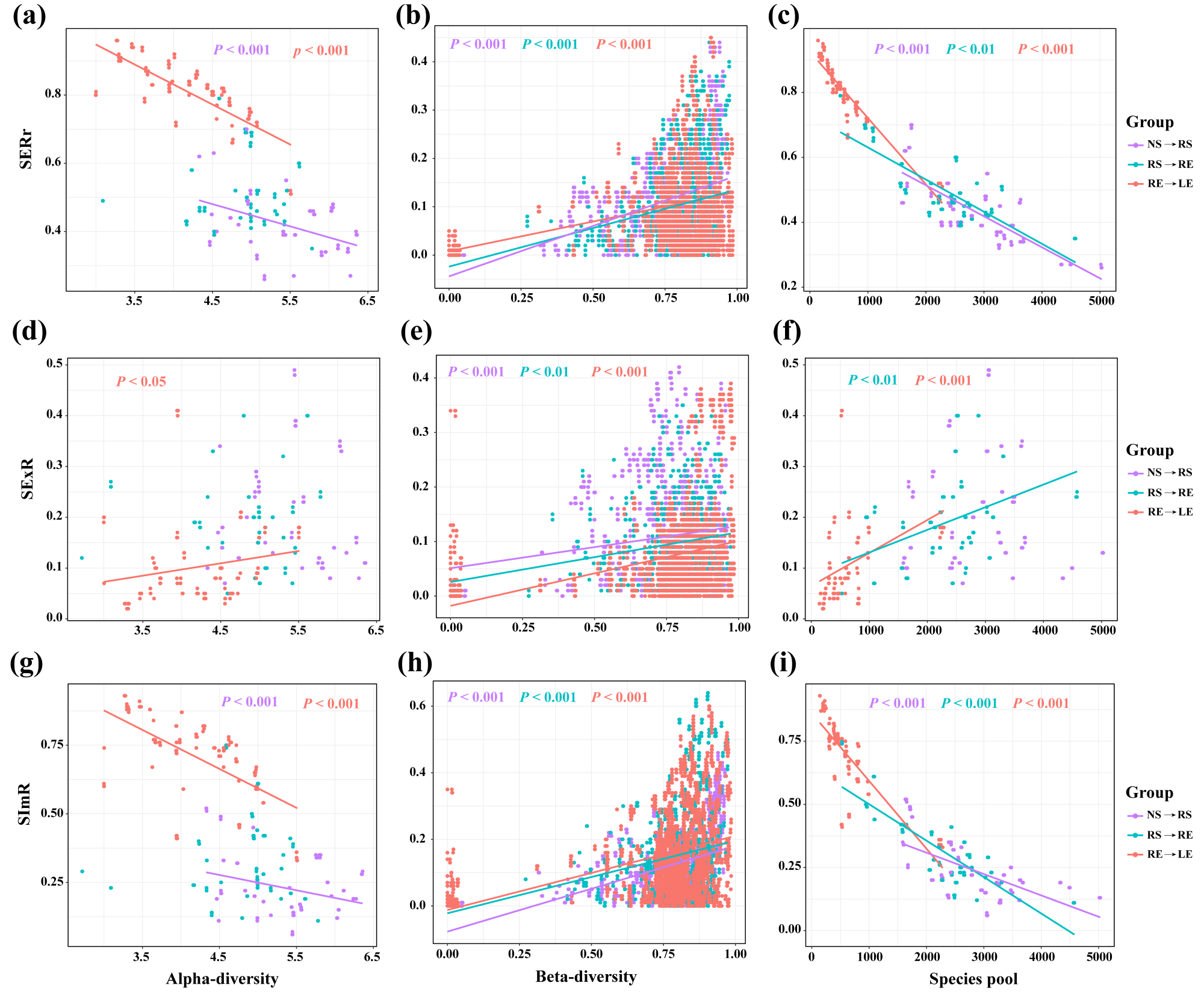


**Fig. S12 The relationships between alpha diversity, beta diversity, species pool, and immigration mechanism of diazotrophic communities.** Linear regression for alpha-diversity, beta-diversity, and species pool associated with species-exchange ratio (SERr) **(a)**, **(b)**, **(c)**, species-extinction ratio (SExR) **(d)**, **(e)**, **(f)**, and species-immigration ratio (SImR) **(g)**, **(h)**, **(i)** across different groups of diazotrophic communities. NS→RS, the transmission pathway from nonrhizosphere soil to rhizosphere soil; RS→RE, the transmission pathway from rhizosphere soil to root endosphere; RE→LE, the transmission pathway from root endosphere to leaves. NS: nonrhizosphere sample; RS: rhizosphere sample; RE: root endosphere sample; LE: leaves sample.

**Table S1 Environmental parameters and geographic location of each plot.**

| **sites** | **Longitude (°N)** | **Latitude (°E)** | **ORP** | **pH** | **Temp (℃)** | **Salinity (‰)** | **Clay (%)** | **Silt (%)** | **Sand (%)** | **TS (%)** | **SO_4_^2-^ (mg/g)** | **TP (mg/g)** | **PO_4_^3-^ (mg/g)** | **TN (%)** | **TC (%)** | **TOC (%)** | **TIC (%)** | **NO_2_^-^-N(μM)** | **NO_3_^-^-N(μM)** | **NH_4_^+^-N(μM)** |
| --- | --- | --- | --- | --- | --- | --- | --- | --- | --- | --- | --- | --- | --- | --- | --- | --- | --- | --- | --- | --- |
| DZ1 | 110.5374 | 20.0016 | -6 | 6.93 | 32.8 | 13.3 | 4.94 | 24.54 | 70.51 | 0.49 | 0.764 | 0.2917 | 0.0687 | 0.1 | 1.9 | 1.79 | 0.11 | 0.833 | 0.151 | 132.356 |
| DZ2 | 110.5374 | 20.0016 | -38 | 6.71 | 30.8 | 20.2 | 6.93 | 32.34 | 60.73 | 0.54 | 0.907 | 0.3616 | 0.061 | 0.094 | 1.8 | 1.62 | 0.18 | 0.417 | 0.244 | 109.159 |
| DZ3 | 110.5386 | 20.0013 | -21 | 6.99 | 30.6 | 21.2 | 3.39 | 20.24 | 76.37 | 0.13 | 0.407 | 0.0362 | 0.0211 | 0.039 | 0.51 | 0.49 | 0.02 | 0.487 | 2.013 | 76.282 |
| DZ4 | 110.5387 | 20.0012 | -5 | 6.89 | 31.9 | 22.8 | 4.32 | 27.39 | 68.29 | 0.2 | 0.401 | 0.2137 | 0.061 | 0.073 | 0.93 | 0.9 | 0.03 | 0.567 | 1.393 | 166.947 |
| DZ5 | 110.5389 | 20.0013 | 3 | 6.98 | 31.8 | 17.2 | 4.06 | 25.24 | 70.7 | 0.18 | 0.505 | 0.8028 | 0.0457 | 0.043 | 0.56 | 0.51 | 0.05 | 0.727 | 1.483 | 80.039 |
| FG1 | 117.9055 | 24.3939 | -51 | 6.9 | 32 | 5.7 | 11.75 | 58.89 | 29.36 | 0.29 | 0.521 | 0.1127 | 0.0814 | 0.163 | 1.84 | 1.68 | 0.16 | 0.387 | 0.76 | 197.143 |
| FG2 | 117.9069 | 24.3940 | 0 | 6.98 | 30.7 | 6.4 | 14.85 | 77.24 | 7.9 | 0.08 | 1.026 | 0.4891 | 0.2374 | 0.132 | 1.21 | 1.07 | 0.14 | 0.51 | 0.406 | 223.192 |
| FG3 | 117.9089 | 24.3945 | -64 | 7.03 | 31.9 | 5.3 | 11.99 | 78.15 | 9.85 | 0.1 | 1.752 | 0.5345 | 0.4898 | 0.134 | 1.32 | 1.27 | 0.05 | 0.234 | 0.101 | 136.908 |
| FG4 | 117.9139 | 24.3945 | -97 | 6.88 | 31.1 | 5 | 14.92 | 62.41 | 22.67 | 0.19 | 0.952 | 0.2052 | 0.1168 | 0.197 | 2.51 | 2.36 | 0.15 | 0.398 | 0.102 | 272.796 |
| FG5 | 117.9204 | 24.3927 | -90 | 6.71 | 29.5 | 8.9 | 11.92 | 55.4 | 32.67 | 0.58 | 1.282 | 0.5264 | 0.2791 | 0.187 | 2.2 | 2.04 | 0.16 | 0.16 | 0.363 | 241.82 |
| HD1 | 114.7679 | 22.8261 | 71 | 7.21 | 34.5 | 19.9 | 3.209 | 20.37 | 76.421 | 0.03 | 5.6 | 0.085 | 0.0014 | 0.115 | 1.51 | 1.44 | 0.07 | 1.04 | 0.295 | 292.034 |
| HD2 | 114.7685 | 22.8267 | 105 | 7.02 | 36.4 | 20.3 | 2.672 | 22.363 | 74.964 | 0.03 | 4.88 | 0.0845 | 0.0014 | 0.102 | 1.44 | 1.38 | 0.06 | 1.04 | 0.242 | 270.278 |
| HD3 | 114.7686 | 22.8267 | 51 | 7.03 | 31.5 | 12.4 | 2.515 | 17.709 | 79.776 | 0.03 | 4.85 | 0.103 | 0.0001 | 0.097 | 1.35 | 1.27 | 0.08 | 0.903 | 0.175 | 241.712 |
| HD4 | 114.7683 | 22.8267 | -48 | 7.06 | 35.6 | 21.3 | 2.292 | 17.011 | 80.696 | 0.03 | 4.2 | 0.06 | 0.0033 | 0.08 | 1.21 | 1.16 | 0.05 | 0.68 | 0.207 | 377.305 |
| HD5 | 114.7680 | 22.8261 | 45 | 7.12 | 36.5 | 19.4 | 3.672 | 22.163 | 74.164 | 0.03 | 4.78 | 0.0828 | 0.0015 | 0.104 | 1.61 | 1.29 | 0.32 | 0.795 | 0.246 | 258.131 |
| LD1 | 108.8525 | 18.4430 | 152 | 8.05 | 32.9 | 22.3 | 8.13 | 52.14 | 39.73 | 0.07 | 0.688 | 0.187 | 0.1169 | 0.052 | 0.66 | 0.57 | 0.09 | 0.678 | 0.195 | 189.208 |
| LD2 | 108.8525 | 18.4430 | 103 | 7.89 | 31.9 | 20.9 | 7.613 | 51.665 | 40.727 | 0.08 | 0.68 | 0.2693 | 0.1401 | 0.028 | 0.5 | 0.36 | 0.14 | 0.589 | 0.218 | 194.289 |
| LD3 | 108.8525 | 18.4429 | 79 | 7.93 | 32.1 | 19.8 | 7.25 | 48.09 | 44.66 | 0.08 | 0.806 | 0.326 | 0.1719 | 0.01 | 0.31 | 0.06 | 0.25 | 0.239 | 0.264 | 102.986 |
| LD4 | 108.8525 | 18.4428 | 32 | 7.31 | 33.1 | 21.6 | 7.29 | 45.77 | 46.94 | 0.1 | 1.12 | 0.3543 | 0.161 | 0.124 | 1.55 | 1.17 | 0.38 | 0.543 | 0.131 | 117.421 |
| LD5 | 108.8525 | 18.4428 | -42 | 6.73 | 29.9 | 14.6 | 8.11 | 48.04 | 43.85 | 0.08 | 1.012 | 0.775 | 0.133 | 0.06 | 0.72 | 0.59 | 0.13 | 1.056 | 0.101 | 122.893 |
| SK1 | 109.6696 | 21.5684 | -9 | 6.98 | 31.2 | 12.7 | 3.071 | 19.943 | 76.986 | 0.09 | 3.24 | 0.068 | 0.0002 | 0.089 | 1.35 | 1.28 | 0.07 | 0.241 | 0.198 | 230.987 |
| SK2 | 109.6697 | 21.5684 | -38 | 6.87 | 32.6 | 13.9 | 2.783 | 25.137 | 72.082 | 0.11 | 3.373 | 0.0551 | 0.0001 | 0.064 | 1.36 | 0.92 | 0.44 | 0.34 | 0.103 | 197.228 |
| SK3 | 109.6698 | 21.5684 | -64 | 6.98 | 31 | 14.7 | 2.187 | 14.091 | 83.722 | 0.11 | 3.38 | 0.029 | 0.0001 | 0.05 | 1 | 0.97 | 0.03 | 0.301 | 0.109 | 161.148 |
| SK4 | 109.6699 | 21.5684 | -90 | 7.03 | 31.8 | 13.9 | 3.624 | 17.409 | 78.972 | 0.13 | 3.387 | 0.0566 | 0.0001 | 0.095 | 1.46 | 1.39 | 0.07 | 0.296 | 0.134 | 184.639 |
| SK5 | 109.6700 | 21.5683 | -29 | 6.93 | 31.9 | 12.5 | 3.88 | 24.341 | 71.779 | 0.11 | 2.57 | 0.067 | 0.0002 | 0.123 | 1.72 | 1.71 | 0.01 | 0.336 | 0.153 | 237.316 |
| ZZ1 | 108.2399 | 21.6188 | -71 | 6.82 | 29.3 | 13.9 | 6.683 | 33.151 | 60.166 | 0.01 | 8.89 | 0.003 | 0.0008 | 0.153 | 2.79 | 2.69 | 0.1 | 0.461 | 0.236 | 717.924 |
| ZZ2 | 108.2399 | 21.6187 | -61 | 6.78 | 29.2 | 16.2 | 2.765 | 35.516 | 61.724 | 0.01 | 7.184 | 0.027 | 0.0005 | 0.152 | 2.93 | 1.98 | 0.95 | 0.213 | 0.335 | 360.315 |
| ZZ3 | 108.2400 | 21.6185 | -81 | 6.74 | 30.4 | 16 | 2.255 | 15.241 | 82.505 | 0.02 | 6.56 | 0.062 | 0.0002 | 0.113 | 2.21 | 1.98 | 0.23 | 0.144 | 0.486 | 249.756 |
| ZZ4 | 108.2401 | 21.6186 | -71 | 6.67 | 30.6 | 16.3 | 2.625 | 23.09 | 74.289 | 0.01 | 7.786 | 0.067 | 0.0002 | 0.154 | 2.8 | 2.15 | 0.65 | 0.197 | 0.392 | 398.92 |
| ZZ5 | 108.2401 | 21.6186 | -77 | 6.68 | 30.7 | 17.3 | 3.095 | 19.16 | 77.745 | 0.01 | 7.73 | 0.054 | 0.0002 | 0.13 | 2.51 | 2.33 | 0.18 | 0.081 | 0.348 | 319.158 |

**Table S1 continued table.**

| **sites** | **Longitude (°N)** | **Latitude (°E)** | **MAT (0.1°C)** | **MAP (0.1mm)** | **MAE (0.1mm)** | **MAR (1%)** | **MAS (0.1h)** | **PTC (%)** | **KN** | **KAH (cm)** | **KBD (cm)** | **KCA (cm^2^)** | **KC (%)** | **KAB (kg)** | **KBB (kg)** | **KTB (kg)** |
| --- | --- | --- | --- | --- | --- | --- | --- | --- | --- | --- | --- | --- | --- | --- | --- | --- |
| DZ1 | 110.5374 | 20.0016 | 258 | 49 | 40 | 80 | 145 | 45 | 6 | 190 | 4.7 | 30000 | 35 | 1.536701 | 1.164819 | 2.70152 |
| DZ2 | 110.5374 | 20.0016 | 258 | 49 | 40 | 80 | 145 | 30 | 5 | 150 | 4.2 | 5600 | 20 | 1.05586 | 0.800342 | 1.856202 |
| DZ3 | 110.5386 | 20.0013 | 258 | 49 | 40 | 80 | 145 | 65 | 8 | 137 | 6.2 | 8000 | 35 | 1.868147 | 1.416055 | 3.284202 |
| DZ4 | 110.5387 | 20.0012 | 258 | 49 | 40 | 80 | 145 | 65 | 4 | 130 | 6.3 | 12000 | 5 | 1.835107 | 1.391011 | 3.226118 |
| DZ5 | 110.5389 | 20.0013 | 258 | 49 | 40 | 80 | 145 | 25 | 1 | 180 | 10.3 | 27000 | 8 | 6.155422 | 4.66581 | 10.82123 |
| FG1 | 117.9055 | 24.3939 | 219 | 31 | 32 | 77 | 54 | 60 | 7 | 600 | 9.6 | 30000 | 30 | 17.37736 | 13.17204 | 30.5494 |
| FG2 | 117.9069 | 24.3940 | 219 | 31 | 32 | 77 | 54 | 60 | 12 | 650 | 10.4 | 45000 | 30 | 22.02984 | 16.69862 | 38.72846 |
| FG3 | 117.9089 | 24.3945 | 219 | 31 | 32 | 77 | 54 | 50 | 12 | 650 | 9.4 | 42500 | 35 | 4.132052 | 3.132095 | 7.264147 |
| FG4 | 117.9139 | 24.3945 | 219 | 31 | 32 | 77 | 54 | 50 | 13 | 280 | 6.7 | 18000 | 30 | 8.917243 | 6.75927 | 15.67651 |
| FG5 | 117.9204 | 24.3927 | 219 | 31 | 32 | 77 | 54 | 65 | 6 | 370 | 8.7 | 45000 | 35 | 22.02984 | 16.69862 | 38.72846 |
| HD1 | 114.7679 | 22.8261 | 222 | 54 | 33 | 78 | 141 | 55 | 5 | 200 | 6.6 | 27000 | 10 | 2.93632 | 2.225731 | 5.162051 |
| HD2 | 114.7685 | 22.8267 | 222 | 54 | 33 | 78 | 141 | 50 | 1 | 210 | 9.3 | 36000 | 2 | 5.866099 | 4.446503 | 10.3126 |
| HD3 | 114.7686 | 22.8267 | 222 | 54 | 33 | 78 | 141 | 50 | 1 | 210 | 8.7 | 36000 | 2 | 5.163019 | 3.913568 | 9.076587 |
| HD4 | 114.7683 | 22.8267 | 222 | 54 | 33 | 78 | 141 | 55 | 1 | 220 | 8.4 | 44000 | 5 | 5.047792 | 3.826226 | 8.874018 |
| HD5 | 114.7680 | 22.8261 | 222 | 54 | 33 | 78 | 141 | 55 | 6 | 200 | 8.2 | 36000 | 10 | 4.40448 | 3.338596 | 7.743076 |
| LD1 | 108.8525 | 18.4430 | 268 | 21 | 55 | 77 | 78 | 5 | 1 | 170 | 5.4 | 15600 | 5 | 1.772332 | 1.343428 | 3.11576 |
| LD2 | 108.8525 | 18.4430 | 268 | 21 | 55 | 77 | 78 | 5 | 1 | 180 | 7.4 | 19500 | 5 | 3.291208 | 2.494736 | 5.785944 |
| LD3 | 108.8525 | 18.4429 | 268 | 21 | 55 | 77 | 78 | 5 | 1 | 160 | 5.7 | 15600 | 5 | 1.847104 | 1.400105 | 3.247209 |
| LD4 | 108.8525 | 18.4428 | 268 | 21 | 55 | 77 | 78 | 5 | 1 | 170 | 7.3 | 15600 | 5 | 3.043983 | 2.307339 | 5.351322 |
| LD5 | 108.8525 | 18.4428 | 268 | 21 | 55 | 77 | 78 | 15 | 3 | 165 | 6.4 | 12000 | 15 | 2.330704 | 1.766674 | 4.097378 |
| SK1 | 109.6696 | 21.5684 | 229 | 46 | 29 | 84 | 217 | 50 | 5 | 210 | 5.6 | 50000 | 25 | 2.277136 | 1.726069 | 4.003205 |
| SK2 | 109.6697 | 21.5684 | 229 | 46 | 29 | 84 | 217 | 45 | 5 | 220 | 5.7 | 28800 | 35 | 2.451418 | 1.858175 | 4.309593 |
| SK3 | 109.6698 | 21.5684 | 229 | 46 | 29 | 84 | 217 | 55 | 8 | 200 | 5.3 | 27000 | 35 | 1.97718 | 1.498702 | 3.475882 |
| SK4 | 109.6699 | 21.5684 | 229 | 46 | 29 | 84 | 217 | 45 | 3 | 210 | 5.7 | 27000 | 35 | 2.350699 | 1.78183 | 4.132529 |
| SK5 | 109.6700 | 21.5683 | 229 | 46 | 29 | 84 | 217 | 40 | 2 | 200 | 5.9 | 27000 | 10 | 2.39382 | 1.814516 | 4.208336 |
| ZZ1 | 108.2399 | 21.6188 | 225 | 41 | 33 | 167 | 135 | 50 | 3 | 270 | 8.8 | 36000 | 15 | 6.717328 | 5.091735 | 11.80906 |
| ZZ2 | 108.2399 | 21.6187 | 225 | 41 | 33 | 167 | 135 | 60 | 2 | 220 | 6.2 | 28800 | 10 | 2.857208 | 2.165764 | 5.022972 |
| ZZ3 | 108.2400 | 21.6185 | 225 | 41 | 33 | 167 | 135 | 50 | 1 | 220 | 4.7 | 36000 | 10 | 1.742138 | 1.320541 | 3.062679 |
| ZZ4 | 108.2401 | 21.6186 | 225 | 41 | 33 | 167 | 135 | 55 | 2 | 230 | 5.3 | 36000 | 10 | 2.238417 | 1.69672 | 3.935137 |
| ZZ5 | 108.2401 | 21.6186 | 225 | 41 | 33 | 167 | 135 | 50 | 5 | 220 | 6.4 | 27000 | 15 | 3.029072 | 2.296037 | 5.325109 |

**Table S2 Mantel tests for the correlation between driving factors and βNTI in the diazotrophic community. Significance levels were indicated: * *P* < 0.05, ** *P* < 0.01, and *** *P* < 0.001.**

| **Driving factors** | **NS** | **RS** | **RE** | **LE** |
| --- | --- | --- | --- | --- |
| ORP | 0.1074 *** | 0.0252 ** | -0.004 | -0.0244 ** |
| pH | 0.1243 *** | 0.0703 *** | 0.0479 *** | -0.044 *** |
| Temperature | 0.1353 *** | -0.0305 ** | 0.1302 *** | -0.049 *** |
| Salinity | 0.0746 *** | -0.0679 *** | 0.0034 | 0.0031 |
| Clay | 0.0045 | -0.0225 * | -0.014 | 0.0357 *** |
| Silt | 0.0147 | -0.0229 * | -0.0225 * | 0.0295 ** |
| Sand | 0.0135 | -0.0231 * | -0.019 * | 0.0311 *** |
| TS | 0.0638 *** | 0.0806 *** | -0.0632 *** | -0.0728 *** |
| SO_4_^2-^ | 0.0571 *** | 0.059 *** | 0.007 | -0.1069 *** |
| TP | -0.0561 *** | 0.0014 | 0.0789 *** | -0.0063 |
| PO_4_^3-^ | 0.0021 | -0.0336 *** | 0.0304 ** | 0.007 |
| TN | 0.0351 *** | 0.0304 ** | -0.0629 *** | -0.0045 |
| TC | 0.0775 *** | 0.0895 *** | -0.0288 ** | -0.0564 *** |
| TOC | 0.0803 *** | 0.0712 *** | -0.0309 ** | -0.0485 *** |
| TIC | -0.0472 *** | 0.0287 ** | 0.0268 ** | -0.058 *** |
| NO_2_^-^-N | 0.0768 *** | 0.0191 * | 0.078 *** | -0.0165 |
| NO_3_^-^-N | -0.0071 | 0.0212 * | -0.0108 | -0.059 *** |
| NH_4_^+^-N | 0.0885 *** | -0.0293 ** | 0.0569 *** | -0.0743 *** |
| MAT | 0.0205 * | 0.038 *** | 0.0037 | -0.0524 *** |
| MAP | 0.0761 *** | 0.0436 *** | 0.0454 *** | -0.0133 |
| MAE | 0.0025 | 0.0543 *** | -0.0049 | -0.049 *** |
| MAR | -0.0278 ** | 0.0774 *** | -0.0427 *** | -0.0632 *** |
| MAS | -0.0485 *** | 0.0071 | -0.0735 *** | 0.0714 *** |
| PTC | 0.0086 | 0.0697 *** | 0.0735 *** | -0.0532 *** |
| KN | -0.0089 | -0.115 *** | -0.0168 | 0.0333 *** |
| KAH | 0.0276 ** | -0.0642 *** | -0.0236 * | 0.0204 * |
| KBD | -0.0608 *** | -0.0085 | 0.0612 *** | -0.0042 |
| KCA | 0.0254 ** | -0.039 *** | -0.0099 | 0.0045 |
| KC | 0.0343 *** | -0.0591 *** | -0.0165 | -0.0167 |
| KAB | 0.032 *** | -0.0349 *** | 0.0469 *** | 0.0443 *** |
| KBB | 0.032 *** | -0.0349 *** | 0.0469 *** | 0.0443 *** |
| KTB | 0.032 *** | -0.0349 *** | 0.0469 *** | 0.0443 *** |

**ORP: Oxidation Reduction Potential**

**Temp: Temperature**

**TS: Total Sulphur**

**TP: Total Phosphorus**

**TN: Total Nitrogen**

**TC: Total Carbon**

**TOC: Total Organic Carbon**

**TIC: Total Inorganic Carbon**

**MAT: Mean Annual Temperature**

**MAP: Mean Annual Precipitation**

**MAE:** **Mean Annual Evaporation**

**MAR:** **Mean Annual Relative Humidity**

**MAS:** **Mean Annual Sunshine**

**PTC:** **Plant total coverage**

**KN: *Kandelia obovata* numbers**

**KAH: *Kandelia obovata* average height**

**KBD: *Kandelia obovata* basal diameter**

**KCA:** ***Kandelia obovata* canopy area**

**KC: *Kandelia obovata* coverage**

**KAB: *Kandelia obovata* aboveground biomass**

**KBB: *Kandelia obovata* belowground biomass**

**KTB: *Kandelia obovata* total biomass**

**References**

1. Wang C, Wang G, Wu P, Rafique R, Zi H, Li X, Luo Y. 2017. Effects of ant mounds on the plant and soil microbial community in an alpine meadow of Qinghai–Tibet plateau. Land Degrad Dev 28:1538-1548. https://doi.org/10.1002/ldr.2681
2. Rahman MM, Zimmer M, Ahmed I, Donato D, Kanzaki M, Xu M. 2021. Co-benefits of protecting mangroves for biodiversity conservation and carbon storage. Nat Commun 12:3875. https://doi.org/10.1038/s41467-021-24207-4
3. Bibi F, Ullah I, Alvi S, Bakhsh S, Yasir M, Al-Ghamdi A, Azhar E. 2017. Isolation, diversity, and biotechnological potential of rhizo-and endophytic bacteria associated with mangrove plants from Saudi Arabia. Genet Mol Res 16:1-12. http:/dx.doi.org/10.4238/gmr16029657
4. Rösch C, Mergel A, Bothe H. 2002. Biodiversity of denitrifying and dinitrogen-fixing bacteria in an acid forest soil. Appl Environ Microbiol 68:3818-3829. https://doi.org/10.1128/aem.68.8.3818-3829.2002
5. Bolyen E, Rideout JR, Dillon MR, Bokulich NA, Abnet CC, Al-Ghalith GA, Alexander H, Alm EJ, Arumugam M, Asnicar F, et al. 2019. Reproducible, interactive, scalable and extensible microbiome data science using QIIME 2. Nat Biotechnol 37:852-857. http:/dx.doi.org/10.1038/s41587-019-0209-9
6. Meng X, Liao H, Fan H, Zhang X, Li Y, Yao H, Razavi BS. 2021. The geographical scale dependence of diazotroph assembly and activity: Effect of a decade fertilization. Geoderma 386:114923. https://doi.org/10.1016/j.geoderma.2020.114923
7. Sun R, Chen Y, Han W, Dong W, Zhang Y, Hu C, Liu B, Wang F. 2020. Different contribution of species sorting and exogenous species immigration from manure to soil fungal diversity and community assemblage under long-term fertilization. Soil Biol Biochem 151:108049. https://doi.org/10.1016/j.soilbio.2020.108049
8. Hillebrand H, Blasius B, Borer ET, Chase JM, Downing JA, Eriksson BK, Filstrup CT, Harpole WS, Hodapp D, Larsen S. 2018. Biodiversity change is uncoupled from species richness trends: Consequences for conservation and monitoring. J Appl Ecol 55:169-184. https://doi.org/10.1111/1365-2664.12959
9. Anderson MJ, Walsh DC. 2013. PERMANOVA, ANOSIM, and the Mantel test in the face of heterogeneous dispersions: what null hypothesis are you testing? Ecol Monogr 83:557-574. https://doi.org/10.1890/12-2010.1
10. Wang Y, Li C, Tu B, Kou Y, Li X. 2021. Species pool and local ecological assembly processes shape the β-diversity of diazotrophs in grassland soils. Soil Biol Biochem 160:108338. https://doi.org/10.1016/j.soilbio.2021.108338
11. Li S, Ren K, Yan X, Tsyganov AN, Mazei Y, Smirnov A, Mazei N, Zhang Y, Rensing C, Yang J. 2023. Linking biodiversity and ecological function through extensive microeukaryotic movement across different habitats in six urban parks. iMeta 2:e103. https://doi.org/10.1002/imt2.103
12. Zhang Z, Pan J, Pan Y, Li M. 2021. Biogeography, assembly patterns, driving factors, and interactions of archaeal community in mangrove sediments. mSystems 6:10.1128/msystems. 01381-20. https://doi.org/10.1128/msystems.01381-20
13. Zhang Y, Hu H, Chen Q, Singh BK, Yan H, Chen D, He J. 2019. Transfer of antibiotic resistance from manure-amended soils to vegetable microbiomes. Environ Int 130:104912. https://doi.org/10.1016/j.envint.2019.104912
